# Modern glucose-lowering drugs in liver transplant recipients: improvement in weight, glycemic control, and potentially allograft steatosis

**DOI:** 10.3389/frtra.2023.1223169

**Published:** 2023-11-30

**Authors:** Srilakshmi Atthota, Kayla Joyal, Mariesa Cote, Riley Scalzo, Ruby Singh, Nikita Consul, Aoife Kilcoyne, Emily D. Bethea, Leigh Anne Dageforde

**Affiliations:** ^1^ Department of Surgery, Division of Transplantation, Massachusetts General Hospital, Boston, MA, United States; ^2^ Department of Pharmacy, Massachusetts General Hospital, Boston, MA, United States; ^3^ Department of Radiology, Massachusetts General Hospital, Boston, MA, United States; ^4^ Department of Medicine, Division of Transplant Hepatology, Massachusetts General Hospital, Boston, MA, United States

**Keywords:** liver transplantation, obesity, metabolic syndrome, antidiabetic medication, weight loss, glucose-lowering medication

## Abstract

**Introduction:**

Recurrent allograft steatosis occurs in one-third of transplanted livers. Antidiabetic agents like glucagon-like peptide-1 receptor agonists (GLP1RA) and sodium-glucose cotransporter type-2 (SGLT2) inhibitors are effective in the management of obesity and hepatic steatosis in the general population; however, there is limited evidence supporting their use in allograft steatosis. We aimed to evaluate their effects on steatosis, body weight, and glycemic control in liver transplant recipients at our institution.

**Methods:**

In this single-center retrospective cohort study of liver transplant recipients currently on a GLP1RA or SGLT2 inhibitor (transplanted 2015–2022), we compared clinical and radiological data before medication use and at follow-up. Differences were compared using Wilcoxon signed-rank test.

**Results:**

Thirty-seven liver transplant recipients were taking the agents. Diabetes was the most common indication (*n* = 33) followed by obesity (*n* = 4). Median follow up was 427 days (301,798). Among those with documented steatosis (*n* = 21), steatosis improved in 5, worsened in 4, remained unchanged in 1, and change could not be evaluated in 11 due to lack of comparable pre and post imaging. Average weight loss was 3.2 kg (*p* < 0.001) and BMI decreased by 1.2 kg/m^2^ (*p* < 0.001). Hemoglobin A1c decreased by 0.6 mmol/mol (*p* = 0.0014), insulin requirement reduced by 7 units/day (*p* = 0.02), and there was no change in additional antidiabetic medications.

**Discussion:**

GLP1RA and SGLT-2 inhibitors are tolerated in transplant patients and result in weight loss and better glycemic control. They are promising agents to treat recurrent or de-novo liver allograft steatosis, but further research is needed to evaluate long-term outcomes in liver transplant recipients.

## Introduction

Non-alcoholic fatty liver disease (NAFLD) is a hepatic manifestation of metabolic syndrome with increasing prevalence globally, parallel to the rise of diabetes mellitus and obesity. It is an increasingly common indication for liver transplantation in the United States (1). Insulin resistance is widely considered to be the underlying mechanism involved in the pathogenesis of these inter-related disorders. Recurrence of allograft steatosis or development of *de novo* steatosis in the transplanted liver is common and occurs in approximately 30% of cases (2, 3). Recurrence rates are higher in patients transplanted for non-alcoholic steatohepatitis (NASH) cirrhosis and are correlated with post-transplant obesity and diabetes mellitus (4). Post-transplant obesity also impacts long-term survival independent of other patient or transplant characteristics (5). A significant number of patients with allograft steatosis also progress to fibrosis; however, the effect on post-transplant outcomes, cardiovascular outcomes and graft survival is unclear (6).

Glucagon-like peptide-1 receptor agonists (GLP1RA) and sodium-glucose cotransporter 2 (SGLT2) inhibitors are modern glucose-lowering antidiabetic agents. GLP1RA improve glycemic control by increasing glucose-dependent insulin secretion and decreasing inappropriate glucagon secretion. They also decrease food intake through delay in gastric emptying, leading to weight loss. SGLT2 inhibitors improve glycemic control by decreasing reabsorption of glucose from the tubular lumen and increasing urinary excretion of glucose. This glucose excretion via the kidneys, also called calorie restriction mimicry, leads to weight loss. Pharmacokinetic and pharmacodynamic properties of GLP1RA and SGLT2 inhibitors are to the advantage of liver transplant recipients since both classes are renally metabolized. GLP1RA are additionally metabolized through glucuronidation. Subcutaneous administration and GI intolerance may affect treatment compliance. There are no theoretical concerns for interactions with tacrolimus pharmacokinetics and several studies showed no difference in serum immunosuppressive drug levels with concurrent use in transplant patients (7–9). In addition to effects of decreased insulin resistance and weight reduction, these agents have various independent mechanisms for alleviation of steatohepatitis such as activation of AMPK-mTOR pathway and TFEB-regulated autophagy-lysosomal pathways, inhibition of NLRP3 inflammasome activation etc., that promote autophagy and inhibit hepatocyte apoptosis (10–13).

These agents were shown to be efficacious in inducing histological resolution of fatty liver disease and improving liver enzymes in the general population (14–17). Several retrospective studies showed that GLP1RA and SGLT2 inhibitors were effective in glycemic control and weight management in diabetic solid organ transplant with no effect on transplant outcomes and immunosuppressant drug levels (9, 18). Their role in management of post-liver transplantation steatosis and obesity has not been well studied. In this study, we hypothesized that liver transplant recipients on GLP1RA and SGLT2 inhibitors would have a reduction in body weight and improvement in graft steatosis.

## Materials and methods

### Study population

We performed a retrospective chart review of all liver transplant recipients who were transplanted between 2015 and 2022 at a single large academic transplant center. We included adult patients who are currently on or have used GLP1RA or SGLT2 inhibitors after liver transplantation and had at least one follow-up visit since initiation of the agent. The study was approved by the institutional ethics review board and informed consent was waived as it was a retrospective review of electronic records. Convenience sampling strategy was used to arrive at study size due to a limited number of transplant patients currently using these agents.

### Data collection

Data on demographics, weight, body mass index (BMI), abdominal ultrasound, Magnetic Resonance Imaging, biopsy, liver function tests, hemoglobin A1c, insulin requirements, medication dosage and adverse effects were extracted from the electronic medical record. Data was collected after liver transplant which was our first timepoint as well as at the most recent transplant clinic follow up.

### Outcomes / definitions

Outcomes of interest are change in body weight, change in degree of graft steatosis, change in hemoglobin A1c and insulin use. Patients with a BMI of 25–29.9 kg/m^2^ were classified as overweight and those with a BMI of ≥30 kg/m^2^ were classified as obese. Previously diagnosed diabetic patients with hemoglobin A1c ≥6.5% were considered to have uncontrolled diabetes. NAFLD Fibrosis score is a composite score calculated using age, BMI, aspartate aminotransferase (AST), alanine aminotransferase (ALT), albumin and platelets. This is a prognostic scoring system that is designed to identify patients at risk for progression of NAFLD to end-stage liver disease (19). Correlated fibrosis severity is drawn from raw NAFLD scores to predict amount of fibrosis. Allograft steatosis was identified on Ultrasound, CT scans, Magnetic Resonance Imaging (MRI)/ Magnetic Resonance Imaging with Elastography (MRE) or available biopsy data. Baseline and follow-up MRI/MRE images, wherever available, were independently reviewed by a single radiologist who was unaware of the clinical status of the patients.

Steatosis was diagnosed and qualified on MRI and MRE examinations using conventional categories of none (<6% fat fraction), mild (6%–17% fat fraction), moderate (17%–22% fat fraction), and severe (>22% fat fraction) on General Electric and Siemens MRI scanners (20). Fat fraction was determined for patients with available MRE examinations directly from the calculated fat fraction map (21). Fat signal fraction was manually calculated on patients with MRI examinations, without available tailored metabolic liver assessment, using the Dixon method on available gradient echo sequences (22).

Donor biopsies were not routinely performed at our institution and only obtained if there is concern for steatosis based on donor clinical history of alcohol use or if there is suspicion of steatotic liver based on gross appearance during procurement. Subsequent biopsies wherever available were performed in liver transplant recipients for the purpose of diagnosing acute rejection, hepatitis or recurrence of disease. Available “for-cause” biopsies with steatosis were reviewed and compared.

### Statistical analysis

Continuous variables were summarized as medians [interquartile range (IQR)]. Categorical variables were summarized with frequencies and percentages. Continuous variables were compared before and after exposure to the medications with Wilcoxon signed rank tests as appropriate for paired samples. In all tests, *P* value less than 0.05 was considered statistically significant. Statistical analyses were completed using STATA version 17.0 (College Station, TX: StataCorp LLC).

## Results

### Demographics

A total of 37 patients who fit the inclusion criteria were identified from the institutional electronic medical record during the 8-year study period. Demographic and clinical characteristics of the study population are summarized in Table 1. Median patient age was 63 years (56, 68) and 22 (59.5%) were male. Using BMI criteria for obesity, 27 (72.9%) patients were obese and 8 (21.6%) were overweight. A total of 20 (54.1%) patients were transplanted for primary or secondary diagnosis of NASH cirrhosis (*n* = 12 with NASH primary diagnosis, *n* = 8 with hepatocellular carcinoma in the background of NASH cirrhosis) (Table 1). Median native Model for End-Stage Liver Disease (MELD) score at transplantation was 23 (11, 30). Donor liver biopsies were available in 21 patients, out of which 11 patients had no steatosis, 9 had mild steatosis: 7 had <5% macrovesicular steatosis, 2 had ∼10% macrovesicular steatosis, and 1 patient had diffuse microvesicular but mild macrovesicular steatosis. In total, 21 (56.7%) out of the entire study population and 11(55%) of the 20 patients with pre-transplant NASH cirrhosis had evidence of allograft steatosis. Out of 21 patients with allograft steatosis, steatosis was detected on imaging in 19 and on pathology in 2. None of the study participants underwent bariatric surgery or used other medical weight loss therapies during the study period. One patient had transferred care from our transplant center and died of an unknown cause. Two other patients died during the follow up period and causes of death were recurrent/metastatic hepatocellular carcinoma and recurrent intrahepatic cholangiocarcinoma.

**Table 1:** Demographics of the cohort.

Baseline Patient Characteristics	*N* (%) or median (IQR)
Age (years)	63 (56,68)
Gender	
Male	22 (59.5%)
Female	15 (40.5%)
Race/Ethnicity	
White	24 (64.8%)
Black	2 (5.4%)
Asian	3 (8.1%)
Hispanic	8 (21.6%)
Other	0 (0%)
Weight class by BMI	
Normal	2 (5.4%)
Overweight	8 (21.6%)
Class I obesity	16 (43.2%)
Class II obesity	6 (16.2%)
Class III obesity	5 (13.5%)
Weight (kg)	96.2 (87.1, 105.7)
BMI (kg/m^2^)	33.2 (29.8, 37.1)
Hemoglobin A1c (mmol/mol)	6.8 (5.7, 8.1)
Insulin requirements (units/day)	32 (0, 55)
Medication Indication	
Diabetes	33 (89.1%)
Obesity	4 (10.8%)
Primary diagnosis at transplant	
NASH cirrhosis	12 (32.4%)
Alcoholic cirrhosis	6 (16.2%)
Hepatitis C cirrhosis	1 (2.7%)
Hepatocellular carcinoma	10 (27%)
Hepatocellular carcinoma + NASH	8 (21.6%)
Pre-transplant metabolic co-morbidities	
Diabetes Mellitus	31 (83.8%)
Hyperlipidemia	17 (45.9%)
Obesity	35 (94.6%)
Cardiovascular disease	7 (18.9%)
Native/ lab MELD at transplantation	
≤9	8 (21.6%)
10–19	6 (16.2%)
20–29	12 (32.4%)
30–39	9 (24.3%)
≥40	2 (5.4%)
Follow-up Data/Medication Data	
Follow-up (days)	427 (301, 798)
Duration of GLP1RA/ SGLT2 treatment (days)	386 (234, 733)
Time since transplant (days)	1,243 (897, 1,571)

### Medications

Diabetes (*n* = 33) was the most common indication for use, followed by obesity (*n* = 4). All diabetic patients were either on other oral antidiabetic medications or insulin at the time of initiation of these agents.Detailed information about medication dosage and type is presented in Table 2. 20 (54.1%) out of 37 patients were using Dulaglutide (Trulicity). Thirty two out of 37 patients were initiated on the medications after their liver transplantation. In the 5 patients that were already using the agents, 4 had no evidence of graft steatosis at any time, and one patient developed mild steatosis of their transplanted liver. Eleven of 37 patients took additional oral antidiabetic agents during the study period. There was not a statistically significant difference in the number of other oral antidiabetic agents before and after initiation of GLP1RA or SGLT2 inhibitors. Average time of follow up after starting medications was 427 days (301, 798) and average time since transplantation was 1,243 days (897, 1,571).

**Table 2:** Summary of antidiabetic medications used by liver transplant recipients in this cohort.

Medication	Number of patients	Common Dosages	Adverse effects	Rates of discontinuation
Glucagon-like peptide-1 receptor agonists				
Dulaglutide (Trulicity)	20	0.75–1.5 mg weekly	Severe gastrointestinal upset (*n* = 2), diarrhea (*n* = 1), decreased appetite (*n* = 1)	2/20
Semaglutide (Ozempic)	6	0.5–1 mg weekly	Suspected pancreatitis (*n* = 1), unknown side effects leading to non-compliance (*n* = 1)	1/6 (suspected pancreatitis)
Liraglutide (Saxenda)	2	1.8–3 mg daily	None reported	-
Exenatide (Byetta)	1	10 mcg daily	None reported	-
Sodium-glucose cotransporter 2 (SGLT2) inhibitors				
Empaglifozin (Jardiance)	5	10 mg daily	None reported	2/5 (stopped due to unstable renal function; unknown reason)
Canaglifozin (Invokana)	1	300 mg daily	None reported	1/1 (therapy completed)
Ertuglifozin (Steglatro)	1	5 mg daily	None reported	-
Dapaglifozin (Farxiga)	1	5 mg daily	Recurrent urinary tract infection (*n* = 1)	-

### Obesity

**Figure 1:**
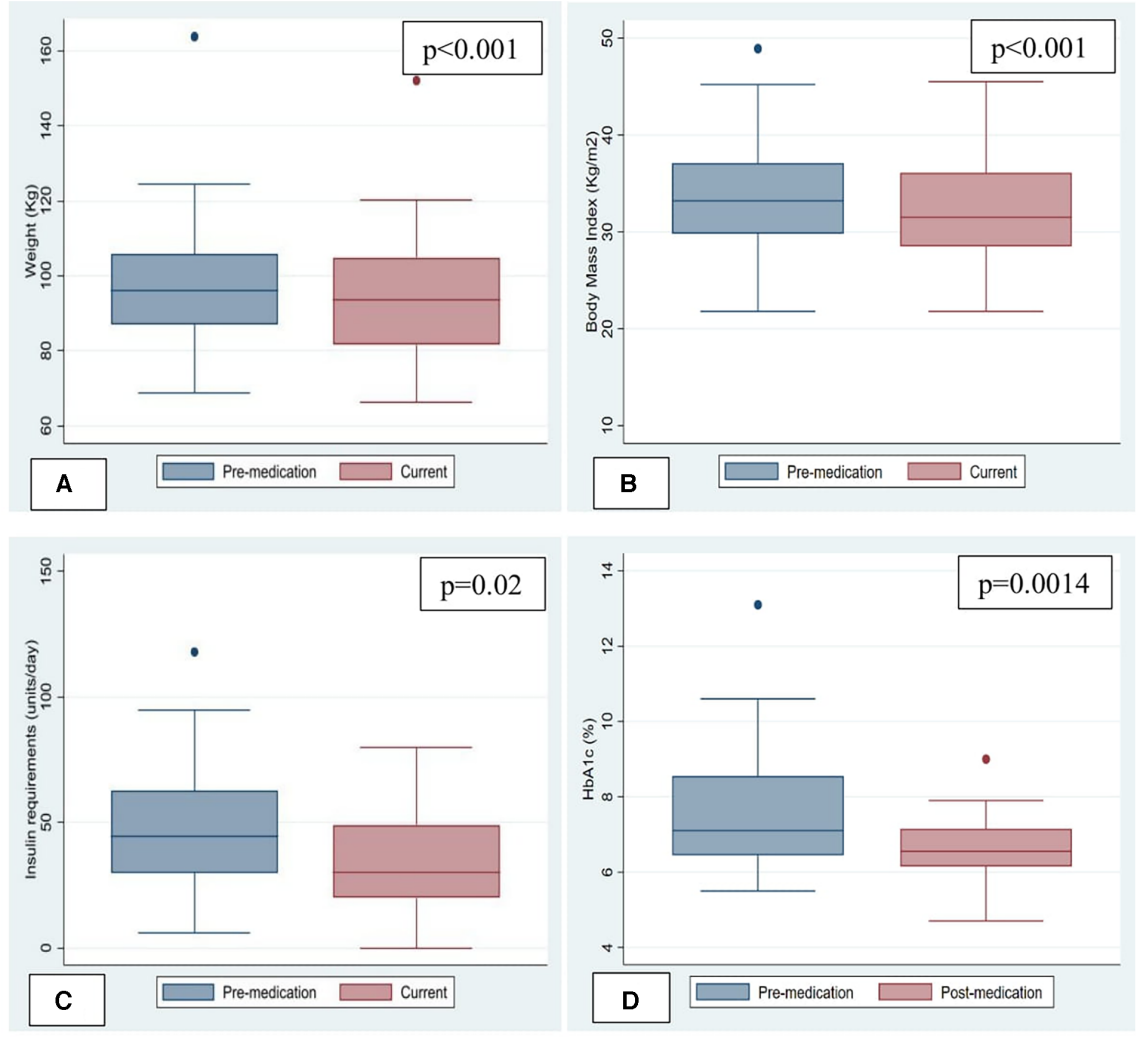
Change in weight, body mass index and metabolic parameters. (**A**) Change in Weight. (**B**) Change in Body mass index (BMI). (**C**) Change in insulin requirements. (**D**) Change in HemoglobinA1c.

Median body weight and BMI at initiation of therapy were 96.2 (87.1, 105.7) kg and 33.2 (29.8, 37.1) kg/m^2^. An average weight loss of 3.2 kg (*p* < 0.001) was noted in the study population when comparing body weight before and after starting the medications. Median weight loss was 3.2 kg (*p* < 0.05) in patients taking GLP1RA compared to 2.8 kg (*p* > 0.05) in patients taking SGLT2 inhibitors. An average decrease in BMI of 1.2 kg/m^2^ (*p* < 0.001) was noted in the study population when comparing BMI before and after starting the medications and this was not significantly different between the two classes (Figure 1).

### Metabolic parameters

Pre-transplantation type-2 diabetes mellitus (T2DM) was present in 31 (83.8%) patients. One patient developed steroid-induced diabetes and another was diagnosed with new-onset T2DM after transplantation. Out of 20 patients who had hemoglobin A1c levels available to compare, 75% had A1c > 6.5% indicative of uncontrolled DM before starting these medications, which decreased to 50% at follow up after starting these medications. Median Hemoglobin A1c at treatment initiation was 6.8 (5.7, 8.1) mmol/mol and decreased by 0.6 mmol/mol (*p* = 0.0014). Median decrease in Hemoglobin A1c was equal with both GLP1RA and SGLT2 inhibitors. 24 out of 33 patients with diabetes were taking insulin. Median insulin requirement at treatment initiation was 32 (0, 55) units/day and decreased by 7 units/day (*p* = 0.02) (Figure 1) In patients taking GLP1RA, median insulin requirement decreased by 7 units/day (*p* < 0.05) however there was no statistically significant difference in insulin requirements in patients started on SGLT2 inhibitors. No significant change was seen in number of additional antidiabetic medications in either group. Subjectively, per documentation by endocrinologists, there was reported improvement in severity of hypoglycemic events on GLP1RA/SGLT2 inhibitors due to the decrease in blood glucose lability in several patients; however, objective data is not available.

### Allograft steatosis

Among those with documented steatosis (*n* = 21), 10 patients have both pre- and post- imaging and biopsy results to compare. The study is underpowered to detect statistically significant differences in grades of steatosis pre/post. While the study is not powered to detect statistical differences, 5 patients had improvement in steatosis once the medications were started, 1 was unchanged and 4 had worsening steatosis (Table 3). Of the 5 that had improvement, all 5 lost weight, a median of 6.4 kg (3.7, 12.4). Of the 4 that had worsening, 1 person gained 1.8 kg and the other 3 lost a median of 6.3 kg. Those who had decreased steatosis had a baseline BMI of 34.2 kg/m^2^ compared to those with worsening of steatosis with a baseline BMI of 30.2 kg/m^2^. The median time on the drug was different between the 5 who improved [473 days (406.5, 704.5)] and the 4 who worsened [578 days (380.5, 736.5)]. There was no significant difference in the degree of donor allograft steatosis in patients who had improvement in steatosis compared to those that had worsening of steatosis. Out of 4 patients with improvement in steatosis, 3 patients were on GLP1RA and one was on SGLT2 inhibitor (Table 3). Out of 7 patients with graft steatosis that were on other oral antidiabetic agents, majority were taking metformin (5 out of 7), one patient was taking glipizide and another patient was on glimepiride.

**Table 3:** Characteristics of steatosis cohort with comparable pre- and post- imaging (*n* = 10). Hepatic fat was quantified by MRI or biopsy(patient #3) Other patients with steatosis have non-comparable modalities (*n* = 4), only pre-treatment imaging (*n* = 6) or have started meds prior to transplant (*n* = 1).

	Agent	Dosage	Duration of follow up (days)	Duration of medication use (days)	Baseline weight (Kg)	Weight change (Kg)	History of pre-transplant NAFLD/NASH cirrhosis	Donor Graft Biopsy—fat fraction	Steatosis degree before initiation of medication	Steatosis degree after initiation of medication	Steatosis change	NAFLD score—Correlated Fibrosis Severity
1	Dulaglutide	0.75 mg weekly	542	661	99.8	−6.4	No	Mild steatosis <5%	Severe	Mild	Improved	Unchanged
2	Dulaglutide	1.5 mg every 2 weeks	705	824	105.7	−13.6	No	No steatosis	Mild	None	Improved	Unchanged
3	Dulaglutide	0.75 mg weekly	626	745	105.2	1.8	Yes	Not available	None	Mild	Worsened	Worsened
4	Dapaglifozin	5 mg daily	392	511	124.5	−4.3	No	Not available	Severe	Moderate	Improved	Unchanged
5	Dulaglutide	1.5 mg weekly	685	804	78.5	−5.9	No	No steatosis	Mild	Severe	Worsened	Unchanged
6	Empaglifozin	10 mg daily	343	462	103	−3.2	Yes	Mild steatosis <10% mixed micro and macrovesicular fat	Severe	Severe	Same degree	Unchanged
7	Dulaglutide	0.75 mg weekly	387	506	112	−3.1	No	Not available	Mild	None	Improved	Unchanged
8	Semaglutide	1 mg weekly	368	487	163.7	−11.7	Yes	Mild steatosis ∼5%	Mild	Severe	Worsened	Unchanged
9	Liraglutide	1.8 mg daily	231	350	78.4	−6.3	Yes	Mild steatosis ∼10%	Severe	Severe	Worsened	Improved
10	Dulaglutide	1.5 mg weekly	264	383	96	−11.2	Yes	No steatosis	Mild	None	Improved	Unchanged

### Hepatic fibrosis

NAFLD Fibrosis score decreased by 0.51 points (*p* = 0.004) and correlated fibrosis severity was not significantly different (*p* = 0.38) in the patients before and after starting the medication. Based on available imaging and pathology reports, no patients were identified to have fibrosis stage ≥F2 in their liver allograft.

### Safety and tolerability

Gastrointestinal disturbances with diarrhea (*n* = 1), decreased appetite (*n* = 1) and severe gastrointestinal upset (*n* = 2) were the commonly reported side effects (Table 2). One patient was presumed to have developed pancreatitis secondary to GLP1RA semaglutide due to close temporal correlation with the start of the medication and the agent was discontinued. One patient reported recurrent urinary tract infections after initiation of the SGLT2 inhibitor Dapagliflozin. Treatment was discontinued in 2 (5.4%) patients due to severe gastrointestinal upset causing non-compliance. Another patient had irregular use due to severe cost prohibition and unstable GFR. Medication was discontinued in two additional patients for unknown reasons. The treatment and follow-up periods were too variable among patients to report changes in immunosuppression regimen or effect of the medications on graft survival. Two patients had biopsy proven mild acute cellular rejection during the follow up period, which was managed successfully with pulse steroids. The data is incomplete for suspected/empirically treated rejection episodes.

## Discussion

Our findings indicate that glucose-lowering medications GLP1RA and SGLT2 inhibitors improve glycemic control, have a weight loss benefit and are generally safe in liver transplant recipients with similar adverse effect profile to the general population. In this retrospective cohort we saw 3.2 kg change in weight for those patients after liver transplantation who were started on SGLT2 or GLP1RA. While only 10 pts had pre/post imaging and biopsy data for steatosis allowing for comparison, half of them showed improvement in steatosis. This was not powered to determine statistical significance, but we did note that those who had improvement had greater weight loss of 6.4 kg and started with marginally higher BMI of 34.2 kg/m^2^ before starting the medication, compared to the entire cohort. However, three out of four patients with worsening steatosis also had a median weight loss of 6.3 kg. There is some data to suggest that presence of greater degree of fibrosis at baseline is associated with lower response rates for treatment (23). None of our patients, including these three, were noted to have greater than stage F2 fibrosis on MRI or for-cause liver biopsies. To our knowledge, there are no independent mechanisms that cause worsening steatosis due to these medications, irrespective of weight change. This unexpected result needs to be evaluated further with adequately powered prospective studies, ideally with matched controls to account for confounders such as other medications, insulin resistance, hyperlipidemia and post-transplant alcohol use.

Other studies have looked at glycemic control and weight change in solid organ transplant recipients on these glucose-lowering medications and our data agrees that there is improvement in glycemic control, a statistically significant weight loss benefit and low incidence of adverse effects (9, 18, 24–27). Several studies found no effect on tacrolimus levels or graft function after initiation of the agents in transplant patients, with one study showing mortality benefit of SGLT2 inhibitors in kidney transplant recipients (9, 26). For steatosis change, while there is evidence in existing literature for improvement in non-transplant patients, the impact on transplanted liver grafts is largely unknown (16, 17). Our data is not powered for statistical assessment, but we did see that 50% of patients had decreased graft steatosis while on these medications. Our study is unique because we present the largest cohort of liver transplant recipients and describe change in steatosis among this cohort of patients. Additionally, we looked at both classes of medications GLP1RA and SGLT2 inhibitors. An ongoing randomized controlled trial to compare glycemic control, body weight, safety, tolerability of oral semaglutide to oral sitagliptin in liver transplant recipients, also incorporates transient elastography at study visits to evaluate change in degree of graft steatosis (28). Another ongoing randomized controlled trial compares effect of dapagliflozin vs. sitagliptin in liver transplant recipients by assessing change in liver and pancreatic fat content as well as change in body composition at 12 months of treatment while maintaining glycemic equilibrium between the groups (29).

This was a retrospective study with a sample size of convenience and thus subject to several limitations including incomplete data and lack of control group. Time points and measurement modalities of steatosis were limited to clinical indications and not universally obtained. We could not correct for confounding and ascertainment biases due to the retrospective nature of this study. Baseline demographics including race, age, BMI and metabolic parameters of patients at our institution may not be representative of general population, making generalizability difficult. Weight was self-reported in some instances due to telehealth visits during the pandemic. Longer follow up is needed to study the impact of steatosis on patient outcomes as the clinical consequences of recurrent graft steatosis do not manifest early. We were not able to account for granular variability in insulin use and hemoglobin A1c measurements due to steroid usage in the patients, as some of the patients were treated empirically with steroids for suspected rejection episodes. We could not comment on the effect of these agents on tacrolimus bioavailability due to lack of data and presence of other potential confounders. In addition, while there is some evidence that sulfonylureas and other classes of anti-diabetic agents may have deleterious effects on steatosis, the number of patients with steatosis in our study that are on these agents is very small, which makes it difficult to draw conclusions on their effect on the progression of graft steatosis.

With the increase in metabolic syndrome, obesity and prevalence of weight gain in liver transplant recipients, management of metabolic complications in this population is becoming important, in addition to management of immunologic, infectious, alcohol use and vascular causes of graft injury. Lack of long-term longitudinal studies studying safety and treatment effect of GLP1RA and SGLT2 inhibitors is a contributing factor to reluctance by regulatory authorities in adapting them as recommended treatment for NAFLD and allograft steatosis. Current published literature relates to the general population and post-kidney transplant patients, which prevents the findings from being extrapolated confidently into clinical practice for liver transplant recipients. Further studies are needed with particular attention to post-liver transplant recipients. Our study and other published studies that report relative safety and tolerability in transplant population can guide future well-designed intervention trials or larger longitudinal prospective studies with clearly defined clinical, radiologic, histologic and transplant outcomes.

These medications have a role in augmenting lifestyle changes for weight loss, including diet modification, exercise, use of connected health technology such as fitness trackers, electronic scales synched to the medical record etc. Multidisciplinary care with a weight center, obesity medicine specialists, transplant pharmacy, nutrition, hepatology and psychosocial team along with use of these medications may improve outcomes. Additionally, for patients with class III obesity, surgical weight loss in combination with medical weight loss assistance could be considered.

## Conclusion

Due to proven benefits in the general population and relative safety and efficacy demonstrated in post-transplant patients, GLP1RA and SGLT2 inhibitors are promising agents to be examined for prevention and treatment of post liver transplant steatosis. Our data highlights the exciting potential that GLP1RA and SGLT2 inhibitors may impact graft steatosis, an increasingly common problem in post-liver transplant patients. In this era of obesity epidemic with increasing rates of NAFLD/NASH as the etiology of end-stage liver disease, further research is required to test the impact of these medications and other novel agents on obesity, metabolic syndrome and post-transplant allograft steatosis.

## Data Availability

The data analyzed in this study is subject to the following licenses/restrictions: We will provide the dataset on request, after obtaining the required institutional permissions. Requests to access these datasets should be directed to ldageforde@mgh.harvard.edu.
